# Growth in Egg Yolk Enhances *Salmonella* Enteritidis Colonization and Virulence in a Mouse Model of Human Colitis

**DOI:** 10.1371/journal.pone.0150258

**Published:** 2016-03-03

**Authors:** Matthew R. Moreau, Dona Saumya S. Wijetunge, Megan L. Bailey, Sudharsan R. Gongati, Laura L. Goodfield, Eranda Mangala K. Kurundu Hewage, Mary J. Kennett, Christine Fedorchuk, Yury V. Ivanov, Jessica E. Linder, Bhushan M. Jayarao, Subhashinie Kariyawasam

**Affiliations:** 1 Department of Veterinary and Biomedical Sciences, The Pennsylvania State University, University Park, PA, United States of America; 2 Department of Food Science, The Pennsylvania State University, University Park, PA, United States of America; 3 Animal Resource Program, The Pennsylvania State University, University Park, PA, United States of America; 4 Animal Diagnostic Laboratory, The Pennsylvania State University, University Park, PA, United States of America; Cornell University, UNITED STATES

## Abstract

*Salmonella* Enteritidis (SE) is one of the most common causes of bacterial food-borne illnesses in the world. Despite the SE’s ability to colonize and infect a wide-range of host, the most common source of infection continues to be the consumption of contaminated shell eggs and egg-based products. To date, the role of the source of SE infection has not been studied as it relates to SE pathogenesis and resulting disease. Using a streptomycin-treated mouse model of human colitis, this study examined the virulence of SE grown in egg yolk and Luria Bertani (LB) broth, and mouse feces collected from mice experimentally infected with SEE1 (SEE1 passed through mice). Primary observations revealed that the mice infected with SE grown in egg yolk displayed greater illness and disease markers than those infected with SE passed through mice or grown in LB broth. Furthermore, the SE grown in egg yolk achieved higher rates of colonization in the mouse intestines and extra-intestinal organs of infected mice than the SE from LB broth or mouse feces. Our results here indicate that the source of SE infection may contribute to the overall pathogenesis of SE in a second host. These results also suggest that reservoir-pathogen dynamics may be critical for SE’s ability to establish colonization and priming for virulence potential.

## Introduction

*Salmonella enterica* serovar Enteritidis (SE) is a Gram-negative, peritrichous, facultative anaerobic, enteric bacterial pathogen. It is the most common cause of salmonellosis in the world making it a major cause of food-borne morbidity and mortality globally [1,2,3,4]. *Salmonella* Enteritidis has an extensive host range and remains an important pathogen for humans and many animal species [5,6]. In the United States alone, the number of SE cases in humans has increased by 44% in the last 20 years [7].

In most cases, SE causes a self-limiting gastroenteritis, which presents with symptoms, such as diarrhea, abdominal cramps, and dehydration. It is not usually fatal although immunocompromised individuals as well as the extremely young or elderly are at high risk for complications of the disease due to systemic dissemination of SE and deficiencies from dehydration [1,5]. If left untreated, these complications can be fatal to these at risk groups. It is estimated that 1:20,000 eggs are contaminated with SE and with approximately 65 billion eggs produced each year in the U.S. alone, about 3.25 million eggs are potentially contaminated. In the 1970’s, surveillance programs and stringent cleaning practices helped to limit the number of illnesses due to egg shell-contamination, however the percentage of internally infected eggs and related illnesses are currently on the rise [1].

Chickens can be born SE infected or acquire SE from the environment via fecal-oral route after hatching [8,9]. Infected hens and rodents, such as mice and rats shed SE in their feces, intermittently contaminating the poultry house environment [10,11]. Rodents are considered one of the biggest risk factors for introduction of SE to poultry and they are likely the source of many plant or produce-based outbreaks of SE [10,12]. Despite the high prevalence of SE in the environment and in animals that are in constant contact with humans, the most important source of SE infections of people remains the shell eggs and egg-based products [13]. Unlike its close relative, *Salmonella* Typhi, there is not much evidence of human to human spread or rodent to human spread through fecal desiccation and dust, which leads to the question of why shell eggs and their products are the major driver of SE transmission to humans. The rise in number of internally infected eggs and human cases suggests a link between the two phenomena. Recent studies have shown that the virulence profile of SE is enhanced when grown inside of an egg. According to a phenotypic microarray, the nutrients within the egg yolk may trigger the expression of certain virulence genes through biochemical linkage [14,15]. Furthermore, a study performed by Gantois and colleagues indicated that growth inside the egg causes SE to express *pef-* fimbriae in *S*. Typhimurium, which has been shown to be important during colonization in the mouse intestines [16,17].

The study presented here was conducted to examine the effect that a particular source microenvironment plays on SE infection in the human host by studying the overall pathogenesis and colonization potential of SE by using a mouse model of human SE colitis, as it relates to the source. This study has far-reaching implications to host-pathogen dynamics in relation to pathogen transmission events and may help us better understand the population genetics, epidemiology, and pathogenic strategies employed by SE and possibly other bacterial pathogens. It will also aid in understanding evolutionary events that allow SE to conserve genes that give it a consistently broad host range, and allow future research to focus on potential eukaryotic targets during the infection process.

## Materials and Methods

### Bacterial strains, and media, and preparation of bacterial innocula

A recently sequenced and characterized SE strain, SEE1 (*S**almonella* Enteritidis isolated from egg; NCBI accession number CP011790), was used in this study [18]. Bacterial inoculations were prepared from SE grown under three different conditions; SE grown in a bacteriologic medium, SE grown in egg yolk, and SE passed through mice. The bacteriologic medium used was Luria Bertani (LB) broth (BD Diagnostics). Bacteria were grown in LB broth overnight at 37°C with continuous shaking. The SE grown in LB broth was subcultured 1:100 into fresh LB broth and grown to an OD_600_ of 1.0. Bacterial pellets were collected by centrifugation at 12,000 xg and resuspended in phosphate-buffered saline (PBS, pH 7.0) (Amresco). To prepare the inoculum of SE passed through mice, mice were first infected with SEE1 after treating with streptomycin as described under “Mouse Experiments and Bacterial Enumeration” section below. Feces from each infected mice was collected separately and aliquots were plated onto xylose lysine deoxycholate (XLD) agar (Hardy Diagnostics) to ensure the presence of SE in the feces. Then, the fecal samples were pooled and organic matter was removed by homogenization by use of Stomacher 80™ bags with a 0.5 mm filter (Seward Limited). The filtrate free of organic matter was used as the inoculum. To grow SE in egg yolk, eggs from organically raised chickens were used. Briefly, egg yolk was separated from albumen and placed in a sterile 50 ml conical tube (Corning). Egg yolk was then inoculated with an isolated colony of SEE1 grown on MacConkey agar (Hardy Diagnostics) and grown overnight at 37°C with shaking. The final inocula were prepared by adjusting the bacterial concentrations in each source to ~10^7^CFU of SE per 50 μl with PBS. To determine the absolute bacterial counts in each inoculum, serial dilutions were made in PBS and plated on XLD agar.

### Mouse experiments and bacterial enumeration

All of the procedures involving mice were conducted as described previously with a few exceptions [19]. Briefly, 6–8 week old C57/B6 mice (Jackson Laboratory) were treated with 20 mg/50 μl/mouse with pharmaceutical grade streptomycin (X-GEN Pharmaceuticals) resuspended in sterile distilled water by oral gavage. This animal model was used because of its susceptibility to many broad host range *Salmonella* species [19,20]. It has been successfully used by other investigators to reproduce bacterial colitis commonly seen during human infection due to SE [20, 21]. Twenty four hours after treatment with streptomycin, groups of at least three mice were then infected with approximately 5x10^7^ CFU of SEE1 grown in LB broth, egg yolk, or mouse feces containing SEE1 by oral gavage. Control mice inoculated with 50 μl of PBS, egg yolk, or feces not containing SEE1 to control for any effects the background media may cause to the mice. Mice were given food and water *ad libitum* except for 4 hours prior to and 2 hours following oral gavaging. The experiment was replicated three times with three to five mice per group. The first two replicates had five mice per group and the last replicate had four mice per experimental group and three mice per control group. Mice were observed for 48 hours post-infection, mortalities were recorded, and surviving mice were euthanized by CO_2_ asphyxiation for necropsy examination and tissue collection. Gross pathological lesions of the organs were recorded and sections of intestines (small intestine, large intestine), liver, and spleen were placed in 10% neutral buffered formalin for histopathology.

Organs were subsequently pooled [by tissue type (liver, spleen, small intestine and the cecum section of the large intestine separate from the colon) and by experimental group], weighed, and placed in Stomacher 80 bags with a filter and homogenized in 1 ml of PBS in a stomacher for bacterial recovery and enumeration. Fecal pellets were collected from each mouse immediately before euthanasia and homogenized in PBS. Serially diluted samples were subsequently plated on XLD agar without antibiotics and plated in triplicate. Black colonies grown on XLD plates were considered as SE and selected for counting. For final statistical analysis, data from three replicates were combined. Therefore, experimental groups contained 14 mice per group and control groups contained 13 mice per group. Data collected were then analyzed using GraphPad Prism Software v. 5.0 (GraphPad Software, Inc.) by either One-Way ANOVA with Tukey’s pairwise comparison post-test for tissues and Kruskal-Wallis with a Dunn’s pairwise comparison post-test for fecal counts as these were taken from individual mice with a significant difference set at *p*<0.05.

### Ethics statement

Animal experiments were carried out in strict accordance with the recommendations in the Guide for the Care and Use of Laboratory Animals of the National Institutes of Health. The protocol was approved by the Institutional Animal Care and Use Committee (IACUC) at the Pennsylvania State University (Protocol Number: 45500). All animals were housed in accordance with the American Association for Laboratory Animal Care standards in groups of up to five animals in individually ventilated cages and kept in a temperature controlled environment (22–24°C) in an animal facility for infected mice. Experiments were planned and conducted utilizing the three R's (reduce, replace and refine) and all efforts were made to minimize animal suffering. Per the IACUC and experimental protocols, the endpoint for each experiment was 48 hours post-infection. If any mice met with two or more clinical symptoms of disease (such as ruffled fur, moribund nature, labored breathing) were to be sacrificed before end of the experiment. However, mice did not show outward signs of disease until 48 hours post-infection. The method of euthanasia was CO_2_ asphyxiation followed by dissection to ensure death. Mice were monitored every 4–8 hours after antibiotic treatment and infection.

### Histopathology

Slides for histopathology were made from formalin-fixed paraffin-embedded (FFPE) sections of the ceca from each mouse at the Animal Diagnostic Laboratory (The Pennsylvania State University). Slides were made with the tissues collected from the last replicate, stained with hematoxylin and eosin (H&E), and viewed with an Olympus BX51 System Microscope (Olympus America, Inc.) at 400x. Twelve to fifteen fields of view for a minimum of three mice per experimental group (four mice per experimental group and three mice per control group) were photographed and analyzed using CellSens Software (Olympus America, Inc.). Total histopathology was scored based on a scoring system developed by Barthel and colleagues [19]. In short, each field was scored on the basis of polymorphonuclear leukocyte (PMN) infiltration of the lamina propria, number of goblet cells, epithelial integrity, and submucosal edema as shown in Table 1. Scores were added, averaged and analyzed by one-way ANOVA with a Tukey’s pairwise comparison test. Statistical significance was considered at *p*<0.05.

**Table 1:** Histopathology scoring system.

Average Combined Score	Interpretation	
0	Intact Intestine without Inflammation	
1–2	Minimal Inflammation and damage	
3–4	Slight Inflammation and damage	
5–8	Moderate Inflammation and damage	
9–10	Profound Inflammation and damage	
**Submucosal Edema**		
**Width**	**Score**	**Description**
0	0	No Pathology
<0.2 mm	1	Mild Edema
0.21–0.45 mm	2	Moderate Edema
>0.46 mm	3	Profound Edema
**PMN Infiltration of Lamina Propria**		
**PMN #**	**Score**	**Description**
<5	0	No Pathology
5–20	1	Mild Infiltration
21–60	2	Moderate Infiltration
>60	3	Profound Infiltration
**Goblet Cells**		
**Goblet Cell #**	**Score**	** **
>28	0	
11–28	1	
1–10	2	
<1	3	
**Epithelial Integrity**		
**Observation**	**Score**	**Morphology/Description**
Normal, continuous columnar cells	0	No Pathological Changes
Sloughing off of epithelium	1	Desquamation
gaps of 1–10 cells	2	Erosion
gaps of >10 cells	3	Ulceration

#### Enzyme-linked immunosorbent assays

Enzyme-linked immunosorbent assays (ELISAs) were performed using DuoSet (R&D Systems) ELISA kits for IFN-γ, IL-1β, and IL-10. All ELISAs were performed using the manufacturer’s instructions in flat bottom 96 well plates (BD Falcon). Pooled cecal homogenate samples from the third replicate (four mice per experimental group and three mice per control group) were diluted 1:2 in PBS and loaded in triplicate for each cytokine. Final optical densities were read using a 450 nm filter on an ELx808 96-well Plate Reader (BioTek Instruments Inc.). Readings were first normalized to the background of the plate. These adjusted readings were used to calculate concentrations of each cytokine in pg/ml based on a standard curve for each cytokine, which were then normalized to the average weight of the ceca of the group. The average cecal weight per group was determined by adding the total weight of the pooled ceca divided by the number of mice in each group. These values were then normalized to the values obtained for their respective controls (formula given below). Data were analyzed by Tukey’s pairwise comparison test following ANOVA and *p*-values<0.05 were considered significantly different.

$$x=\Sigma \left((\frac{B}{A}(D))-C\right)/n$$

X = Average pg of cytokine

B = pg cytokine/mL

A = Average cecal weight of the group

D = Dilution factor

C = Average control [cytokine]

N = Number of mice in the group

*Note $\left(\frac{B}{A}(D)\right)-C$ is to calculate individual sample [cytokine]

### Gross pathology and biometrics

Photographs of mice and ceca from the third replicate (four mice per experimental group and three mice per control group),were taken using a D5200 Nikon digital camera at the time of necropsy to determine the gross pathological lesions. Ceca were placed on a graph paper in comparison to a ruler in order to measure cecal size differences among different treatment groups before taking the photographs. Percentage weight change of the mice was calculated by the formula below. Mice were ear punched to keep track of the individual mice and the weight of each mouse was recorded at the beginning of the experiment (after streptomycin treatment, prior to infection).

$$\text{x}=\Sigma \left(((\frac{\text{B}}{\text{A}})*100)\text{-C}\right)/\text{n}$$

x = Average % Body weight change

B = Change in body weight

A = Original body weight

C = Percent body weight change average of control

N = number of mice in the group

*Note $\left((\frac{B}{A})*100\right)-C$ is to calculate % body weight change per mouse

## Results

### *Salmonella* Enteritidis grown in egg yolk displays enhanced colonization of the mouse intestines

**Fig 1:**
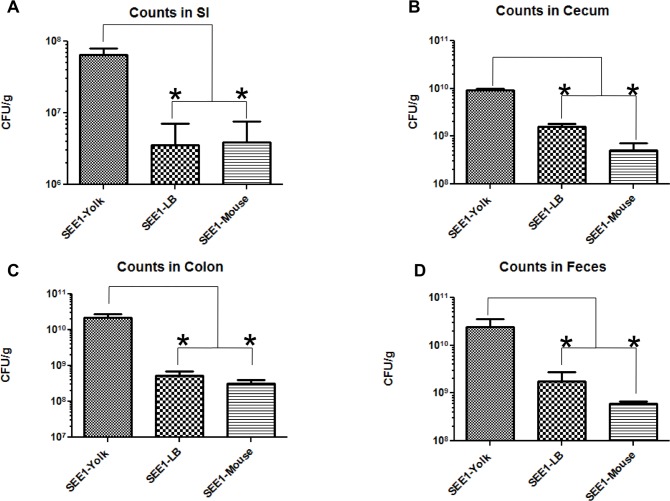
Bacterial burden in the intestinal tract and fecal shedding. Above are the results of the bacterial burden in the mouse intestines after infecting with SEE1 from different sources. Bacterial counts of the small intestine (A), cecum (B), colon (C) as well as fecal shedding differences (D) were taken from three experiments, normalized with corresponding controls, and the results analyzed by one-way ANOVA with a Tukey’s post-test (A-C) or Kruskall-Wallis test (D) and * represents *p*<0.05. There were five mice per group in the first two experiments and four mice per group in the last experiment. Mice that received the same treatment in each of the three experiments were considered one group for final data analysis.

We first sought to establish if the growth medium or source of SE would have an effect on its ability to colonize the mouse intestines. In a streptomycin-treated mouse model of SE-induced colitis, SEE1 grown in all three sources (egg yolk, mouse feces, and LB broth) were able to colonize the mouse intestines (Fig 1A, 1B and 1C). The degree of colonization of the small intestine by SE was much less than in the cecum and colon. The mice infected with SEE1 grown in egg yolk displayed a greater colonization in the mouse intestines compared to the groups infected with SE in mouse feces or SE grown in LB broth. The difference in each of the intestinal tissues was approximately one log-fold or greater between the groups infected with SEE1 grown in egg yolk and SEE1 from the other sources. No significant difference was observed between the groups treated with SEE1 grown in LB broth and SEE1 from mouse feces.

### *Salmonella* Enteritidis grown in egg yolk increases fecal shedding

Due to the increase in GIT colonization observed for SEE1 grown in egg yolk, we next probed for differences in ratios of fecal shedding. There was an increase in fecal shedding (CFU/g) of SEE1 from mice infected with SEE1 grown in egg yolk compared to the other two sources (Fig 1D). Similar to the difference observed in the colonization of the intestines, the group infected with SEE1 from egg yolk demonstrated at least 1-log-fold difference in shedding compared to SEE1 grown in LB broth or from mouse feces.

### SEE1 grown in egg yolk shows variable increase in dissemination

**Fig 2:**
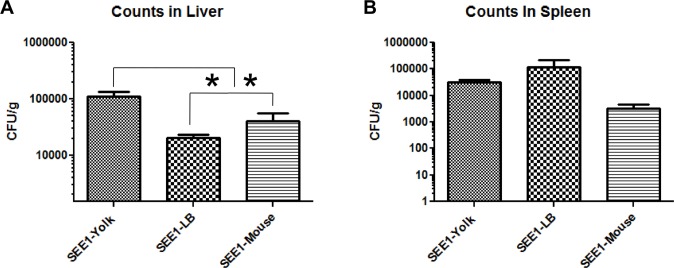
Extra-intestinal dissemination of SEE1. Bacterial burden of the liver (A) and spleen (B) from three experiments was taken and normalized with corresponding controls, and analyzed by Tukey’s post-test of one-way ANOVA, and significance of *p*<0.05 is indicated by *. There were five mice per group in the first two experiments and four mice per group in the last experiment. Mice that received the same treatment in each of the three experiments were considered one group for final data analysis.

*Salmonella* Enteritidis has the ability to disseminate to extra-intestinal sites, such as the liver and spleen, and thus we determined if the source of SEE1 had an influence on its extra-intestinal dissemination. SEE1 grown in egg yolk was found to colonize the mouse liver to a significantly greater degree when compared to SEE1 from the other two sources, although the difference was less than 1-log (Fig 2A). There was no statistical significance in colonization of the spleen among SEE1 grown under the three different conditions examined in this study (Fig 2B).

### Growth in egg yolk enhances SEE1 virulence *in vivo*

**Fig 3:**
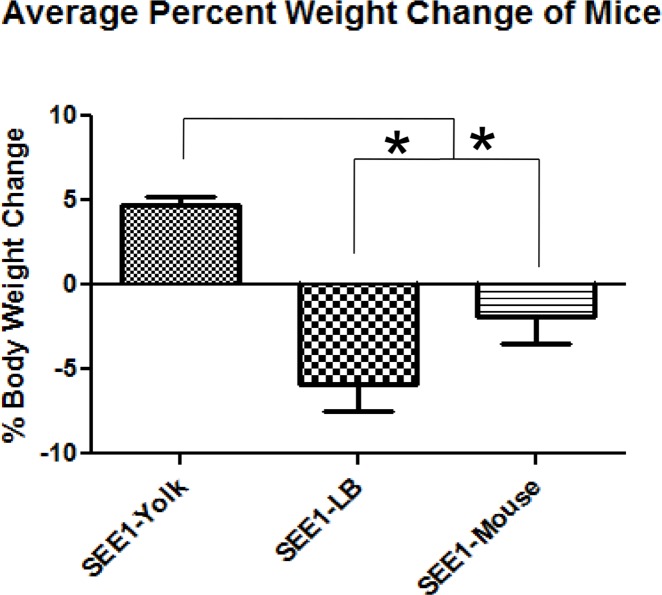
Percent weight changes in mice. The overall weight of mice is an indicator of gastrointestinal distress of mice. Mice from the third experiment (four mice in each experimental group and three mice in each control group) were weighed at the beginning and end of the experiment with SEE1 from various sources and the difference was compared to respective controls. The weight changes were taken from at least two experiments, normalized with corresponding controls, expressed as percent weight changes, and analyzed by Tukey’s pairwise comparison test following a one-way ANOVA. Significance was considered for *p<*0.05 and shown as *.

Mice infected with SEE1 from yolk showed more serious symptoms of disease and were more likely to be moribund than SEE1 from LB broth or feces. Mice infected with SEE1 grown in egg yolk retained more body weight than the mice infected with SEE1 from two other sources (Fig 3). To further study differences in pathogenesis between groups, biometric markers of inflammation and infection were measured.

**Fig 4:**
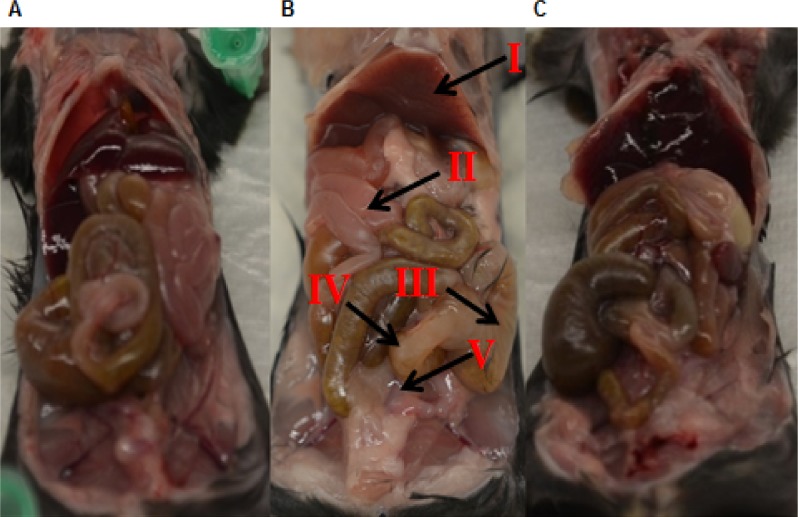
Gross pathological changes in mice. Overall changes in the mice internal organs from the third experiment were photographed and analyzed. Mice infected with SEE1 grown in egg yolk (B) appear to have greater outward pathology than the mice infected with SEE1 from LB broth (A) or from mouse feces (C). Major changes in gross pathology and organs of interest are indicated by the arrows and Roman numerals. I, The liver displaying marked paling, and prominent blood vessels. II, The small intestine showing signs of paling compared to the small intestine in A and C. III, The cecum that is emptied and shriveled. IV, The colon section of the large intestine that is extremely pale and empty. V, Fluid build-up in the visceral tissues. There were four mice in each experimental group and three mice in each control group.

**Fig 5:**
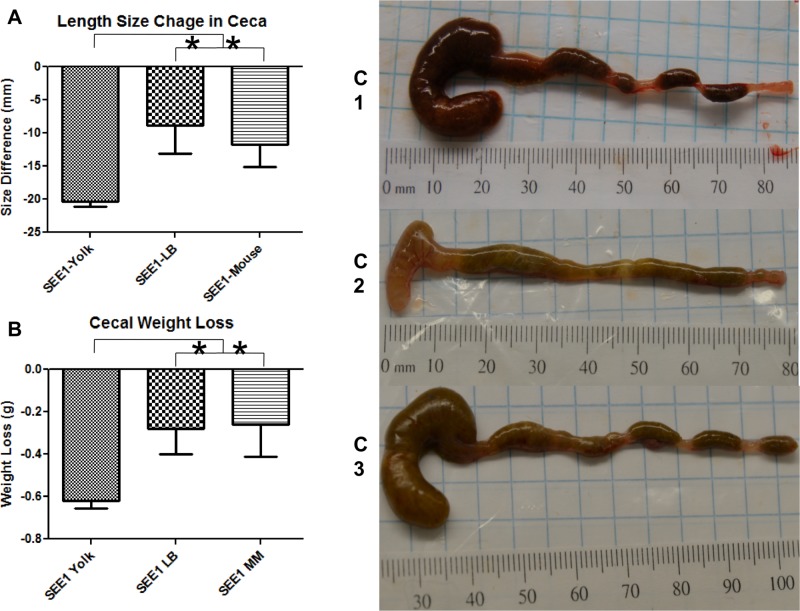
Biometrics of the ceca. The overall size (A), weight (B), and pathology-driven morphological changes (C1-C3) were determined for the mice infected with SEE1 from the three sources. There were four mice in each experimental group and three mice in each control group. Measurements of the ceca from the last experiment were taken and normalized with corresponding controls, yielding the changes in size and weight. SEE1 grown in egg yolk appears to cause greater pathological changes in the ceca as compared to SEE1 grown in LB broth (C1) or passed through mice (C3). For A and B, significance was determined by one-way ANOVA Tukey’s pairwise comparison test, and significance was set at *p<*0.05; indicated by *.

Distinct changes were observed in the gross pathology of the internal organs, with the most striking differences seen in the ceca and liver. Mice infected with SEE1 from yolk displayed signs of inflammation (whitish color edema and prominent blood vessels) in the liver, whereas livers of the mice in other groups appeared healthy (Fig 4). Similarly, the ceca of the mice infected with SEE1 from yolk were extremely pale, shriveled, and emptied; while mice in other experimental groups had enlarged ceca with signs of mild inflammation (Fig 4 and Fig 5C1–5C3). This observation was further supported by the overall dimensional changes of the ceca. On average, ceca infected with SEE1 from egg yolk were significantly lighter (by weight) and smaller (in length) compared to ceca from the other two infected groups (Fig 5A and 5B). In both the ceca and the liver of the mice infected with SEE1 grown in egg yolk, blood vessels were very prominent indicating severe inflammation at these sites. Similarly, most of the viscera of the peritoneal cavity displayed signs of inflammation (edema and paling) as well as evidence of toxemia and increased perfusion.

**Fig 6:**
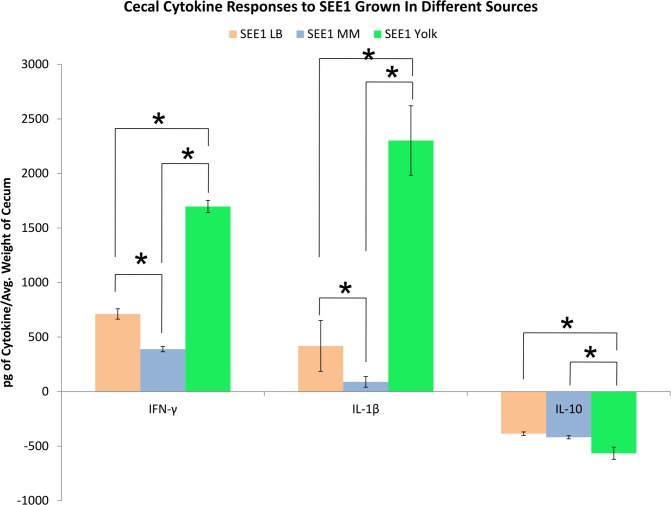
Cytokine analysis of ceca of mice infected with SEE1 from three sources. Pro-inflammatory and anti-inflammatory cytokine profiles had been determined by ELISA for ceca of mice infected with SEE1 from egg yolk (four mice), LB broth (four mice), and passed through mice (four mice) as well as corresponding controls for each source used (three mice each). Results were normalized with corresponding controls and analyzed through one-way ANOVA and subsequent Tukey’s Pairwise Comparison Test. Significance of *p*<0.05 is indicated by *.

**Fig 7:**
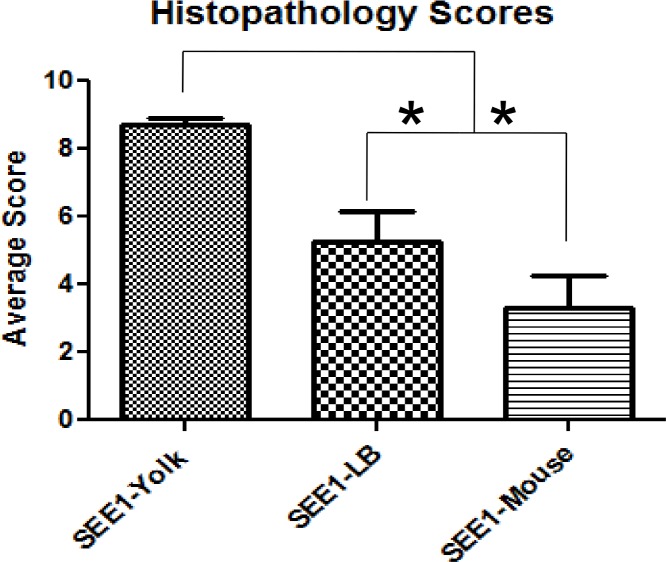
Overall histopathology scores of ceca. The primary site of colonization for SE in mice is the cecum; so the histopathology of the ceca of three mice from each control group and four mice from each infection group was determined. Statistical significance was determined by one-way ANOVA Tukey’s pairwise comparison test, and significance was set at *p*<0.05; indicated by *.

**Fig 8:**
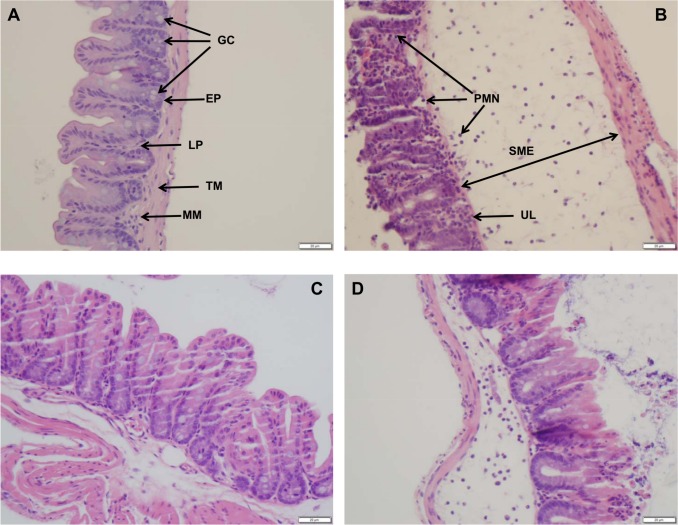
Changes in the histopathology of ceca infected with SEE1 from various sources. This Fig shows a representative micrographs of sections of a healthy ceca from an uninfected mouse (A), and cecal sections of mice infected with SEE1 grown in egg yolk (B), SEE1 passed through mice (C), and SEE1 grown in LB broth (D). Abbreviations: GC (Goblet Cells), EP (Epithelial Cells), LP (Lamina Propria), TM (Tunica Muscularis), MM (Muscularis Mucosa), PMN (Polymorphonuclear leukocytes), SME (Sub-mucosal Edema), UL (Ulceration).

To associate the inflammatory changes seen in the cecum to inflammatory response, cecal homogenates were subjected to cytokine analysis by ELISA to test for levels of interferon gamma (IFN-γ), Interleukin-1 beta (IL-1β), and interleukin-10 (IL-10). As shown in Fig 6, the levels of IL-1β and IFN-γ were significantly higher in the ceca of mice infected with SEE1 from yolk in comparison to other groups. This change correlated to the suppression of IL-10. With the increases in gross pathology and biometrics in the mice infected with SEE1 from yolk, cellular damage was assessed using histopathology. Ceca from individual mice were sectioned and scored based on a previously defined scoring system. By quantitative (Fig 7) and qualitative (Fig 8) microscopic analysis of these sections revealed that SEE1 from yolk resulted in more lesions and more severe pathology in mice at the cellular level than in mice infected with SEE1 from LB broth or mouse feces.

## Discussion

*Salmonella* Enteritidis remains a constant threat to human and animal health [7,13, 22]. For humans, the predominant cause of bacterial-foodborne morbidity and mortality is non-typhoidal *Salmonella enterica* infections, of which SE is the most common [22]. Despite the fact that SE can infect a wide range of host species and evidence that humans acquire SE from a wide-range of vectors, shell eggs and egg-based products remain the primary vehicles of transmission of SE to humans [5,9,13]. Why shell eggs and egg products remain the most common source of SE-associated foodborne illness has yet to be understood from the standpoint of pathogen transmission dynamics and disease pathogenesis. To our knowledge, there has never been a study that has probed the effect of pathogen source on SE virulence and pathogenesis.

As a non-typhoidal *Salmonella enterica* serovar, SE primarily causes gastrointestinal disease but can disseminate to the liver and spleen through the lymphatic system [23]. Studies conducted using a streptomycin-treated mouse model of *Salmonella* colitis have revealed that for non-typhoidal *Salmonella enterica* serovars, such as Typhimurium and Enteritidis, the primary site of colonization is the cecum, but they can also be recovered from the other intestinal sites, extra-intestinal sites, and lymphatic tissues [19,24]. The ability of SE to colonize its host and cause disease is determined by genes located primarily on five major pathogenicity-associated islands (PAI) though some virulence factors are located outside of these PAI [25]. Among the five major PAIs, *Salmonella* Pathogenicity Islands 1 and 2 (SPI1 and 2) are essential for the ability of SE to colonize and spread [5].

For a Gram-negative bacterial species like SE, initial colonization is crucial. The site of SE colonization creates a microenvironment rich in LPS, toxins, and other effectors as a result of collateral damage via pro-inflammatory cytokines and neutrophils [26]. Because bacterial gene expression is determined by its environment, the source of SE may have an effect on SE virulence in the subsequent host. This study in fact demonstrated for the first time that the source of SE influences the subsequent disease pathogenesis in the host. The mouse intestines had significantly higher colonization by SEE1 from yolk than SEE1 from LB broth or passed through mice. The fact that SEE1 grown in egg yolk also had colonized the liver to a significantly greater extent as compared to SEE1 from the other sources also meant that the microenvironment of egg yolk increased the virulence of SE including its colonization and invasive abilities perhaps by upregulation of respective virulence genes. Other evidence in support of this hypothesis is shown by the degree of damage to the organs in mice infected with SEE1 from egg yolk as compared to the mice infected with SEE1 from LB broth or feces. The organs of mice infected with SEE1 from egg yolk have increased signs of inflammation and necrosis; most notably in the cecum (primary site of colonization for SE) and the liver (primary site of disseminated colonization for SE) as shown in Figs 4 and 5. The quantitative and qualitative histopathological lesions of the cecum were in consistent with the gross pathological observations as evidenced by a significant increase in the overall inflammation of the cecum in mice infected with SEE1 from egg yolk as compared to SEE1 from other two sources. Inflammation and cellular damage is more severe and widespread in these mice compared to those infected with SEE1 grown in LB broth or passed through mice.

Mice infected with SEE1 from yolk also displayed several histopathological markers consistent with increased virulence. The ceca showed more erosion and ulceration of the epithelium, including loss of epithelial integrity as compared to mice in other experimental groups. Additionally, PMN infiltration of the lamina propria of the cecum was pronounced. Of note, however, was that mice infected with SEE1 that was passed through mice showed significantly less histopathological lesions compared to SEE1 from egg yolk or LB broth in spite of no difference in colonization abilities. Differences in microscopic and macroscopic pathology between groups correlated with cytokine expression. All SE infected mice irrespective of the source of SE were able to stimulate production of pro-inflammatory cytokines, such as IL-1β (in response to LPS) and IFN-γ (in response to intracellular pathogens) compared to uninfected controls. Just like the damage and inflammation that was seen previously, SEE1 from yolk elicited a stronger pro-inflammatory cytokine response as well as a strong suppression of the anti-inflammatory cytokine IL-10. However, SEE1 from LB broth or feces was attenuated in their cytokine response as compared to SEE1 from yolk.

Taken together, these results suggest that the source of an SE infection plays a significant role in the pathobiology of that host-pathogen interaction, resulting in significant differences in overall disease outcome. It can be inferred that these changes most likely occur at the point of SE transmission leading to a change in the disease kinetics rather than a continuous change over the course of the infection. This is because at the time of transmission, SE’s transcriptional program will be the same as it was in the yolk and later during the infection this program will adapt to the microenvironment of the host. It is worth noting that these data support the novel finding that SE passed through mouse is attenuated in virulence compared to SE from the other two sources. This is in contrast to what has been seen previously with other *Salmonella enterica* serovars as well as other enteric Gram negative bacteria such as *Vibrio cholerae* where inter-species passage actually increases colonization and virulence [27].

Of the five major PAIs found within SE, SPI-1 is essential for invasion and initial colonization, and SPI-2 is essential for dissemination and macrophage survival [5]. Both SPIs are regulated by a number of well-known signal response systems including PhoP/Q, LsrR, QseB/C, and oxygen tension [28–31]. The SPI-1 is also co-regulated with SPI-4 [32]. We hypothesize that the high cell density of SE (10^9^CFU/ml) will activate the autoinducer-1 (AI-1) and subsequently, the AI-2 systems in combination with low oxygen levels in egg yolk will upregulate the expression of SPI-1, 2, and 4 prior to transmission. Egg yolk is extremely nutrient-rich and likely supplies the energy required by SE to express all of the genes on these PAIs in response to these signals [28].

In the egg yolk, SE might become pre-programmed to cause infection in the human host due to the synthesis of the PAIs’ gene products, including two type III secretion systems and a plethora of effectors prior to colonization. This allows for quicker colonization, invasion, and initial damage prior to adapting genetically to the host, resulting in more severe lesions in the colonization sites like those seen in cecal histopathology. An increase in SPI-1expression in turn increases the level of the NF-κB antagonist AvrA, allowing the bacteria to colonize and invade the tissues without initial recruitment of inflammatory cells at the site of colonization [33]. Once in these sites, these bacteria will downregulate SPI-1 and upregulate SPI-2, the mediator of many SE intracellular survival traits [34]. This allows the infected cells to upregulate NF-κB regulated pro-inflammatory cytokines and recruits PMNs to the sites of infection. The PMN infiltration and subsequent oxidative burst damage the intestinal epithelial cells and microbiota releasing ethanolamine and tetrathionate into the lumen [35,36]. In this microenvironment, SE can dominate because it is protected against the oxidative burst by its superoxide dismutases and can use ethanolamine and tetrathionate to drive anaerobic respiration [37]. This increase in initial colonization followed by subsequent events would explain the pronounced differences in damage seen in these mice from SEE1 grown in egg yolk as well as the strong pro-inflammatory response (by way of increase in pro-inflammatory cytokines as well as decrease of anti-inflammatory cytokines) in response to bacterial burden differences.

Altogether, this study illustrates two very important and novel findings related to microbial pathogenesis and specifically to SE pathogenesis. The first is that the source or reservoir of a bacterial pathogen can influence the outcome of disease in a new host. The second is that the increase in colonization and virulence of SE in mice after being grown in egg yolk may explain why shell eggs and egg products are the most common vehicle of SE transmission. More work must be done to explore the underlying mechanisms that SE uses to undergo this transcriptional programming event. More importantly, examining the relationship of the source-bacteria-host epidemiological triad is very important in understanding the overall disease pathogenesis.
